# Return to Work of Breast Cancer Survivors: Perspectives and Challenges for Occupational Physicians

**DOI:** 10.3390/cancers12020355

**Published:** 2020-02-04

**Authors:** Marcello Campagna, Roberto Loscerbo, Ilaria Pilia, Federico Meloni

**Affiliations:** Department of Medical Sciences and Public Health, University of Cagliari, Blocco I, SS 554, km 4,500, 09042 Monserrato, Italy; mam.campagna@gmail.com (M.C.); roberto.loscerbo@gmail.com (R.L.); cromatinap@gmail.com (I.P.)

**Keywords:** breast cancer, return to work, fitness for work

## Abstract

Breast cancer is one of the most common diseases worldwide, mainly affecting the female gender. Considering the increase of breast cancer incidence and the decrease of mortality due to news diagnostic and therapeutic tools, the return to work issue after treatment is going to be very common in the next years. Occupational physicians therefore need to face the return to work and the fitness for work of workers previously diagnosed with breast cancer with a sufficient cultural and technical background. In addition to individual characteristics preceding the diagnosis, clinical outcome, lifestyles and occupational variables are the most impactful factors on return to work that need to be taken into account. The aim of this work is to analyze these factors and discuss the central role of occupational physicians in the decision-making process of returning to work in breast cancer survivors.

## 1. Introduction

Breast cancer (BC) is one of the most common diseases worldwide. The estimated incidence in 2018 was ≥72.9 cases per 100,000 person years and the mortality rate was about 14 cases per 100,000 person years in developed countries; in the same year the age standardized rate of incidence in North America was 84.8 cases per 100,000 person years, while in Northern Europe it was 90.1 and in Southern Europe 80.3. In the same period and areas, the age standardized mortality rates were 12.6, 14.1 and 13.3 per 100,000 person years, respectively [1]. In Italy, breast cancer is the most common cancer in women: in 2019, 53,000 new cases have been estimated and in 2016 BC was the leading neoplastic mortality cause among females (13,000 deaths) [2].

During the last 40 years, diagnostic tools have improved more than ever, leading to a huge increase in diagnosis, especially in situ, during screening programs, which could explain the incidence of increase. The chances of an early diagnosis offer the opportunity to eradicate the disease. These medical progresses have contributed to reducing mortality, especially in those countries capable of offering the best quality care [3,4]. Globally, in 2002 there were 8 million cancer survivors worldwide, of whom 22% were BC survivors. By 2022, it is estimated that this number will be close to 18 million; in addition, about 70% of cases occur in working age adults [5]. 

Considering the increase of breast cancer incidence, the age at the time of the diagnosis and the decrease of mortality, the return to work (RTW) issue after surgery is going to be very common in the next years. For this reason, those impactful factors that could get in the way of return to work or ease it should be discussed. Some studies showed average time to RTW: form 11.4 months in the Netherlands, 7.4 in Canada, to only 3 months in Sweden. Percentages of RTW after 1 year ranged from 54.3% in a cross-sectional study from France to 82% in a prospective study from the USA [6]. These results are the product of multiple factors that we can resume in three macro topics: clinical outcomes, lifestyle and occupational variables. These factors make RTW process a long path and the patient should be assisted from the beginning by occupational physicians (OPs) [7].

On the other hand, OPs can play a major role in the prevention of BC in terms of primary prevention (by reducing exposure to specific occupational risk factors and encouraging female workers to follow healthy lifestyles), in secondary prevention through tailored screening strategies that take into account both occupational and individual risk factors, and in tertiary prevention, promoting rehabilitation, thus also facilitating the RTW. 

## 2. Discussion

The evaluation of a patient returning to work after a breast cancer curative treatment should consider all the three aforementioned factors:

- The clinical outcomes, in order to know what to expect from a subject who has been diagnosed and treated for a BC, better address the fitness for work (FFW) and eventually promoting a tertiary prevention by specific rehabilitation programs;
- Lifestyles, in order to promote or modify those that may or may not facilitate the return to work, thus applying primary prevention even in the RTW process;
- Occupational variables, in order to know tasks performed and occupational risks to better address RTW and FFW choices.

Clinical outcomes can be divided in six main aspects: arm’s range of movements, muscular strength, pain, lymphedema, cognitive impairment and psychological wellness and changes in activities of daily living. 

The range of movement of the homolateral arm mostly depends on three factors: type of surgery, presence/absence of axillary lymph nodes dissection (ALND) and presence/absence of radiotherapy. Mastectomy showed an increased risk in the reduction of the range of motion (ROM) five times higher than lumpectomy [8]. Additionally, ALND and sentinel node biopsy increase the reduction of ROM [9]. Similar results were obtained when subjects that received radiotherapy were compared with those who did not [10]. Level I evidence was found in a systematic review for mastectomy and radiotherapy to the axilla as risk factors for reduced ROM in abduction, flexion and external rotation and level II evidence for ALND and radiotherapy to the chest wall [11]. The same systematic review found level I evidence for ALND and concurrent radiotherapy and chemotherapy as risk factors for reduced muscle strength [11]. Research on radiation induced upper body morbidity has uncovered a wide range of issues, including skin fragility, fibrosis and inflammatory changes to the soft tissue in the irradiated area, as well as brachial plexopathies and other neuropathic impairments that may lead to sensory and motor changes [12].

The level I evidence risk factors for pain are: ALND, radiotherapy before chemotherapy and administration of zoledronic acids. Level II evidence was found for the sentinel node biopsy and radiotherapy as risk factors for pain. Arm and shoulder pain has an important influence on low quality of sleep; furthermore, chemotherapy alone is a risk factor for pain [11]. The level I evidence risk factors for lymphedema are: ALND, radiotherapy to the axilla, radical mastectomy, concurrent radiotherapy and chemotherapy [11].

Cognitive impairment, including problems with concentration, attention, memory, multitasking, executive functioning, speed of processing and decision making, are crucial for returning to work. Those patients with higher levels of subjective cognitive impairment were more likely to be unemployed, to have left the workforce or have lower work output [13]. Psychological wellness after diagnosis and treatment for BC also has a significant impact on RTW. Many patients could present depression, fatigue, worry, frustration and fear of potential environmental hazards [14]. On the other hand, in a recent systematic review about functional impairments and Work-Related Outcomes in Breast Cancer Survivors, authors suggested that “findings show that physical functioning was univocally related to RTW and work ability, whereas findings for other domains of functioning were not as straightforward. This might partly be because in scientific literature, the concept of work disability is primarily focused on physical aspects of functioning, and to a lesser extent on cognitive, social and emotional aspects. This might be reflective of what happens in practice. Indeed, occupational health physicians evaluating disability in cancer survivors have reported to rely mainly on a biomedical approach, while subjective complaints of psychosocial functioning, which are harder to assess, take a less prominent position [15].

Changes and reduction in activities of dialing living in BC survivors were observed [16]. This scenario could also be matched with a possible reduction in ability in work activities. OPs should therefore investigate and take them into account, using this information in RTW and FFW process. In this regard, the use of specific questionnaires and scales may be useful (e.g., Katz’s index of activities of daily living—ADL) [17], as well as advice from occupational therapists [18,19].

The kind of treatment should always be considered; a Swedish study found that 35% of 72 women studied did not return to work after 12 months and 9% did not return after 18 months, assuming that chemotherapy and multimodal treatment might be the issue. Still, overall quality of life in their patients improved between the 8th and 11th months after BC diagnosis, independently of the type of adjuvant treatment [20].

Regarding lifestyles, smoking and drinking are the most important preventable risk factors. A higher risk was found in women who started smoking before their first pregnancy and in long term and in heavy smokers (>40 packs/year). Alcohol showed a dose-dependent relationship; a consumption of 3–6 glasses of wine per week has a 10% increased risk. Additionally, binge drinking showed an increase in risk [21]. The mechanism behind these findings is likely related to the ability of alcohol to increase levels of estrogen in the blood [22]. On the other hand, fitness and nutrition have a very important protective role: breast cancer risk is significantly decreased in women performing regular physical activity and in those who consume high amounts of fruit and vegetables. Women who walk at least 7 hours per week have a 10–25% lower risk of BC compared to women who are inactive, with an even greater protective effect in postmenopausal women [23]. Furthermore, these women tended to have less aggressive cancers. Risk is approximately 1.5 higher in overweight postmenopausal women and 2.1 higher in obese postmenopausal women. This is likely explained by higher levels of circulating estrogen released from adipose tissue in elderly women [24]. 

The role of age is also important, approximately 99.3% and 71.1% of all breast cancers associated deaths were reported in women over the age of 40 and 60, respectively [25]. Additionally, reproductive factors such as early menarche, late menopause, late first pregnancy and low parity can increase breast cancer risk [26]. Each 1-year delay in menopause increases the risk of breast cancer by 3% [27]. Each 1-year delay in menarche and each additional birth decreases the risk of breast cancer by 5% and 10%, respectively [28].

Younger age, higher education, marital status single, high income and positive social support from friends and family increase the likelihood of an early return to work. On the other hand, a lower educational level, low household income, overprotective family and poor social support represent barriers for return to work among cancer survivors [14]. According to some authors, primary reasons to return to work are financial pressure, fear of being sacked and being too old for a new job [13].

The practice of regular exercise before the diagnosis could increase self-esteem and confidence in physical abilities, which may increase the chances of RTW. Furthermore, practicing regular exercise before a diagnosis of breast cancer could enhance the capacity to perform activities of daily living during treatment and can facilitate a return to pre-illness fitness following treatment [29].

One of the main aspects to consider in occupational variables is the task performed: manual workers, compared with non-manual workers, have decreased chances of employment [30]. In a German study, 66% of patients returned to work, 16% of those who returned to their previous job reported disadvantages, with a higher rate for manual than for non-manual (23% vs. 7%) [31]. 

Another relevant aspect is working in the public or private sector: in the former, employees have more chances to maintain their job compared to the latter [30].

Other relevant aspects influencing the chances of being employed after breast surgery could be timing and seniority: senior employees and employees in the public sector are more likely to fully return to work later than junior employees and employees in the private sector [32]. During the first weeks of returning to work, the psychological perception of the workplace (job facility, flexibility and support from colleagues, as well as the perception of job importance) can motivate cancer survivors to work. On the other hand, stressful jobs, lack of support from employers, colleagues and OPs, reduced working hours and decreased wages are factors identified that discourage survivors to re-enter their jobs [14].

After the period of sick leave, gradually starting back to work again combined with a trajectory of learning new skills can support patients to overcome the negative side effects of treatment, restore confidence and give room to adapt to altered circumstances [30]. Employer support, defined as all possible interventions made by employer to facilitate the vocational rehabilitation of the employee, was positively associated with RTW [33]. At the same time, a greater company size usually means a bigger variability of tasks and the possibility to offer a small number of “low stress dedicated jobs”. This kind of organization could support RTW, even in more delicate cases [34]. Additionally, the type of employment (full-time or part-time) is an occupational variable that could affect the RTW. In a Japanese study, the median duration of sickness absence until either partial or full time RTW was 80 days, the median time to full RTW among breast cancer survivors was 209 days. Women returned to work part time 4.8 times more compared to those returning to full time employment [35].

An Italian study reported a duration of sick leave less than 3 months in 55.4% of cases, but longer than six months in 23.9%. About 43% of the women reported reduced work ability upon returning to work. Women with a reduced work ability were more likely to consult an OP (48.4% vs. 31.6%) and the OP cooperated with the employer to find work adjustments in 69.9% of cases. However, women with reduced work ability reported less support from their employer, colleagues and more discrimination. Eventually, the probability of work disability increases as time off for illness increases [36].

A Swedish study analyzed the relationship between the impact of healthcare professionals on RTW: 80% of women had experienced the encounter “advice and support related to work”. Of these, 57% of women with high stage at diagnosis were recommended to be on sickness leave, while 70% of I and II stage patients had been encouraged to work. Furthermore, those that had experienced encounters regarding work form healthcare professionals had fewer days of sick absence during the first two years compared to women who seldom/never experienced such encounters (35.2 vs. 44.9 days). Eventually, those women who were always/often encouraged to work had fewer sick days absence compared to those had seldom/never experienced such encounters (31.5 vs. 48.1 days) [37]. In this regard, multidisciplinary approaches have already been proposed to improve the RTW of patients with cancer. A multidisciplinary rehabilitation program, which combined occupational counselling with a physical exercise program supervised by a sports physician and a physiotherapist during chemotherapy, resulted in increase of rates of RTW, in reduced fatigue and increased importance of work, work ability and quality of life. Occupational counselling was provided by an oncological OP [38], who is an OP with a specific training relating to the support of patients with cancer who encounter work-related problems [38,39,40].

In this context, the role of OPs is crucial, using their knowledge to inform patients about the advantages of rehabilitation programs, healthy lifestyles and facilitations guaranteed by the law. A good OP should dialogue with oncologists, physiotherapists, psychologists and occupational therapists to better understand and investigate the clinical outcomes that affect the RTW and FFW. OPs should cooperate with the employer to find work adjustments, if necessary and play a major role in individual risk assessment. However, the OP is not always ready to face this challenge. Shim et al. studied the experiences of occupational physicians on cancer survivors’ return-to-work. Only 25% had experience regarding cancer survivors, including workers with previously diagnosed with BC [41].

As well as the aforementioned aspects that affect the RTW, OP should use these macro categories of factors during the decision-making process of the FFW in BC survivors:

- neoplasia features (age at diagnosis, histology, staging, prognosis);
- worker’s global health (comorbidities, radiotherapy/chemotherapy, positive psychological sensations about his tasks, work ability and will to work);
- work features and related occupational risks (night shift work, exposure to ionizing radiations, polycyclic aromatic hydrocarbons, ethylene dioxide, biomechanical overload upper limbs, job complexity and organizational aspects);
- laws and guidelines.

All these factors are irreplaceable and will help OP in the FFW decision-making process.

These macro categories were proposed by Taino et al. for judgment of FFW in workers exposed to ionizing radiations and with a history of cancer disease [42]. Taino et al. considered a macro category positive if the majority of the single voices was positive. A possible and similar strategy could be proposed for RTW in workers previously diagnosed with BC. A low risk profile would be defined if neoplasia features criteria, worker’s global health, work features and laws and guideline are negative or only one of them is positive. In this case a low risk profile does not need specific adjustments and can consider a patient fit for work. An intermediate risk profile would be defined if 2/4 of macro categories are positive, and in this case, a limitation or a prescription in order to protect our patient should be considered. A high-risk profile would be defined if three or four macro categories were positive. In this case workers could be in danger and health workers should probably suggest that the employer allocated new tasks [42]. Every situation is unique and should be considered an individual merit. OPs should dialogue with employers, trying to find a customized RTW schedule (including more appropriate tasks) with the aim of motivating employees and showing them flexibility [43].

On the other hand, OPs could play a major role in terms of prevention. 

First of all, they should give advice on primary prevention; in this case our objective is to quantify individual risk attributable to a worker and his job with the aim of reducing the risk of a primary BC and relapses. OPs should discern between changeable and unchangeable breast cancer risk factors. 

Changeable risk factors are tobacco smoke, alcoholic beverages, high postmenopausal body mass index, sedentary lifestyle, estrogens, occupational exposure to night shifts, ethylene dioxide, pesticides, acrylic fibers, polycyclic aromatic hydrocarbons and ionizing radiations [26,44,45]. Healthcare professionals should encourage patients by a valid health promotion to stop smoking and drinking, start taking regular physical activity and follow a healthy diet and prescribe estrogens only when necessary, explaining that each preventive measure is fundamental in protecting from cancer and improving the quality of life. A new individual risk assessment should be mandatory after a cancer diagnosis, evaluating both the presence of night shifts, ethylene dioxide, pesticides, acrylic fibers, polycyclic aromatic hydrocarbons and ionizing radiation. Those risks should be avoided or carefully quantified, in order to protect workers from a new illness [42,44,45].

Among the unchangeable risk factors, the most important is age, followed by reproductive factors such as early menarche, late menopause, late first pregnancy, low parity, BRCA1/BRCA2 gene mutations and other genomic polymorphisms [26]. About 20–25% of hereditary cancers and 5–10% of all breast cancers are caused by BRCA1/2 mutations [46]. These unchangeable factors have to be considered during individual risk assessment because they represent a dangerous additional burden.

OPs should encourage a secondary prevention by BC screening in female workers at risk of BC and in BC survivors. The BC screening program is highly recommended, especially for those that have a high-risk profile, which includes having been previously diagnosed with BC. The greatest reduction in BC mortality after the introduction of screening programs was observed in women of 60–69 years old (RR 0.69; 95% CI: 0.57–0.83); nonetheless, this model is useful from 50 to 74 years of age. The subscription to mammography screening can influence the stage at diagnosis, for this reason we observed a decrease in rate of mastectomies and an increase in the rate of breast conserving surgery [47]. For these reasons, the BC screening program is strictly connected to RTW because an early diagnosis is often followed by a better recovery and improved work ability. Occupational physicians should therefore always take BC screening into account, stratifying workers into risk categories: workers who have not yet been diagnosed with BC; workers who have not yet been diagnosed and with occupational risk factors for BC; BC survivors without additional occupational risk factors for BC; BC survivors with previous additional occupational risk factors for BC.

Finally, tertiary prevention in those workers with clinical outcomes, also with the help of physiotherapists, psychologists and occupational therapists [18,19,38,48,49].

In this context, it would be necessary to increase and better define the role of OPs in the health promotion process. As part of the health surveillance of millions of people, OPs should have a strategic role in the prevention of chronic non-communicable diseases, in support the adherence to screening programs and in promoting correct lifestyles. These goals are already plan and promote in some countries. For example, in Italy in 2018 the Ministry of Health signed a memorandum of understanding together with the president of Italian Society of Occupational Medicine in relation to these topics [50]. 

## 3. Conclusions

Scientific evidence suggests that a multidisciplinary approach would be preferred. A good OP should liaise with oncologists, physiotherapists, psychologists, occupational therapists and employers. Each one of these figures should cooperate, offering a unique point of view, in order to help overcome physical and relational disfunctions [7,18,19,38,43,51].

Occupational health professionals should take note of individual and collective risk assessment, promote a healthy lifestyle before and after sick leave, encourage rehabilitation and propose solutions with the aim of improving the interactions between employees and workplace [52].

Encouraging patients with low stage diagnosis returning to work could be useful, improving their quality of life, reducing days of sick leave and requests of disability pension. High stage patients could need more time to recover from chemotherapy; however, they should be encouraged if their general conditions allow for it [37]. 

RTW and FFW process should take into account all the elements described and use the risk profile considering also worker’s desires and fears. Despite limited information on those who have had to change their job position after cancer diagnosis [53], return to work should symbolize a return to normality and social reintegration [54] representing a challenge for the OPs that can enhance their key role within the workplace and in society. As suggested by other authors, more evidence is required regarding safety, health or ergonomic interventions, for manual workers and their RTW needs and findings for domains of functioning other than physical [15,53]. 
